# Facing the National Recovery and Resilience Plan: Sources of Data, Indicators, and Participatory Strategies in Healthcare and Social Fields

**DOI:** 10.3390/ijerph181910457

**Published:** 2021-10-05

**Authors:** Michela Franchini, Sabrina Molinaro, Michelangelo Caiolfa, Massimiliano Salvatori, Stefania Pieroni

**Affiliations:** 1 Institute of Clinical Physiology, National Research Council, 56124 Pisa, Italy; sabrina.molinaro@ifc.cnr.it (S.M.); msalvatori@ifc.cnr.it (M.S.); stefania.pieroni@ifc.cnr.it (S.P.); 2 Federsanità-Anci Toscana, 50122 Firenze, Italy; m.caiolfa@gmail.com

**Keywords:** health promotion, community-engaged research, community-based participatory research, community empowerment, NRRP, participatory tools

## Abstract

Innovation in governance and services should be the target of the Italian National Recovery and Resilience Plan. Monitoring processes, impacts, and outcomes requires a system of new indicators that are practical to collect. Secondary data sources, their availability, and their information potential should be evaluated, and primary sources should be implemented to supplement traditional disease surveillance. This work highlights the most relevant aspects for bridging the mismatching between complex community needs and current health/social supply and how those aspects could be faced. As a result, we propose a structured multi-phases process for setting the design and functionalities of a cooperative information system, built on the integration between secondary and primary data for informing policies about chronic low back pain (CLBP), a widely recognized determinant of disability and significant economic burden. In particular, we propose the Dress-KINESIS, a tool for improving community capacity development and participation that allows one to freely collect big health and social data and link it to existing secondary data. The system also may be able to monitor how the resources are distributed across different care sectors and suggest how to improve efficiency based on the patient’s CLBP risk stratification. Moreover, it is potentially customizable in other fields of health.

## 1. Introduction

The National Recovery and Resilience Plan (NRRP), which outlines how Italy will invest €191.5 billion from the European Union to emerge from the pandemic crisis, has been submitted to the European Commission.

60% of these resources are loans that should be paid back by generating public value. This implies that during the next 5 years, the Italian Government put in place mechanisms that reward implementation, and not just good intentions, for achieving specific aims both in the social and health field, according to the definition of innovation not just as a new idea but a new practice that produces incremental changes [1].

Innovation in governance and services is usually not a physical artefact but a change in the relationships between service providers and their users. In such changes, judgements have to be made about processes, impacts, and outcomes, as well as the products. Innovative approaches have to be identified through cooperative (policy maker, public manager, and population), multilevel (innovation at both central and local level), and evidence-based supported (research and scientific networks) approaches, built on a large system of indicators monitoring the knowledge, behaviors, or practices that the programs or interventions intend to change [2]. The purpose of monitoring is to provide routine, timely information for program management on whether the program is making progress towards meeting its objectives. Selecting appropriate indicators (input, output, and outcome indicators) is the previous step in outcome monitoring: indicators play a central role in the monitoring process by generating regular and objective feedback about progress towards policy objectives.

Regarding the NRRP strategies, output and outcome indicators should play the most relevant role, considering that the availability of resources will depend on the achievement of specific goals, appropriately scheduled throughout the 5-year process of implementing planned actions. So, planned policies have to produce effective outputs (what a policy produces directly with the inputs that are provided) that influence the outcome (why a policy should be implemented) to produce both economic and social development at the Italian national level. In other words, it is necessary that the policy objectives clearly distinguish between what a policy produces and what are the reasons for implementing the policy.

Moreover, the Italian NRRP provides for a considerable amount of investment in the field of physically immaterial outcomes, such as the improvement of the public organization’s performance through human capital development, digital transformation, and a deep revision of the organizational processes. This makes the Italian plan particularly challenging regarding the identification of effective methods of planning; monitoring; and evaluations. This also implies that indicators need to be specific to the population and program they are monitoring, as well as considering regional and local variability and organization skills.

Additionally, indicators should be practical to allow routine collection and be responsive to program effects. To support the process of monitoring, program planners must decide on information needs (if secondary data sources can be used or whether primary data collection is necessary), and the frequency with which outcomes will be measured. They must also understand the information environment in which the programs will be operating, including knowing what types of data are already being collected in the target area, how often the data are collected, and their quality. The program must assess the resources, such as money, time, and staff effort; they can allocate for monitoring purposes, including possible primary data collection.

In particular, the sixth NRRP Mission, “Healthcare”, allocating a total of € 15.6 billion for strengthening the Italian healthcare system, includes two components: (1) proximity assistance and telemedicine, and (2) healthcare innovation, research, and digitalization [3]. The healthcare mission is in line with the “one health” approach, supported by the European Commission, which provides for the wide use of digital leverage by: the sharing and use of eData-Big Data; machine learning, AI, and deep data analysis technologies; the e-Care and connected-care projects; and the evolution of the electronic health record towards the citizen’s file.

For these aims, two reference paradigms should be considered: Benington’s and Hartley’s competing paradigm, named the “citizen-centred governance”, or “networked governance”, [4] which assumes a larger role of the public institutions as co-producers of service and innovation, and the Greenhalgh’s statement [5], which suggests that, for the National Health Service, innovations have to be “perceived as new by a proportion of key stakeholders”. 

**Figure 1:**
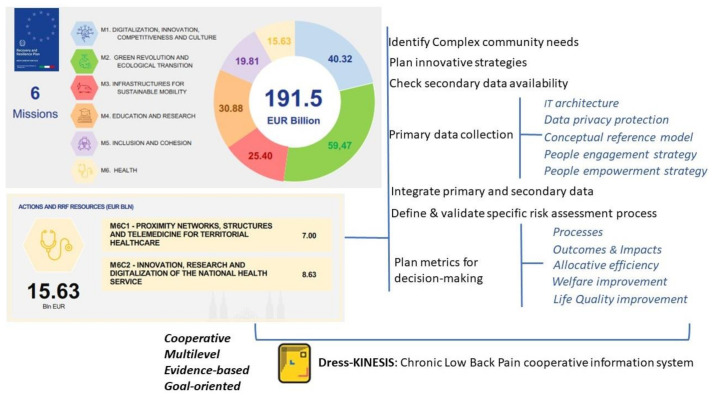
Article topics.

According to this perspective, our work aims to highlight the most relevant aspects for bridging the mismatching between complex community needs and the current health/social supply and how these aspects could be implemented to build innovation in governance and services, which is particularly relevant for NRRP implementation (Figure 1). 

In particular, this paper describes: (1) the secondary sources of data already existing in Italy in health and social fields, their main characteristics, and their information potential; (2) the participatory paradigm in primary data collection; (3) the availability of innovative tools for freely collecting big data exploiting the combined technology offering and its widespread access across all segments of the population; and (4) their potential role for promoting people engagement, community knowledge, and empowerment, and informing public policies.

As a result, we propose a structured multi-phases process for setting the design and functions of a cooperative information system, built on the link between secondary and primary data. The process is a feasibility study focused on chronic low back pain, potentially customizable in other fields of health. 

Low back pain (LBP) is a symptom affecting 28.8% of people, 39.0% of whom are men and 60.9% of whom are women [6], and it is ranked as the first cause of disability among 291 conditions and sixth in terms of overall burden in the Global Burden of Disease 2010 study [7]. Only about 40% of people with back pain will seek medical advice, and around 10% of such patients will have significant disability, with a subset attending the Emergency Department [8]. Among patients suffering from acute LBP, 5–10% [6] will develop persistent or recurrent low back pain (chronic low back pain), considered to be a chronic condition with recurrent symptomatic episodes [9].

Chronic low back pain (CLBP) determines high economic burden both in terms of direct costs (medications, hospitalizations, and outpatients), indirect costs (loss of productivity, disability-adjusted life years), and intangible costs (reduced enjoyment of life, including the inability to interact with others). A recent study in the Netherlands [10], performed on 1502 patients (age 46.3 ± 12.8 years, 57% female), reported mean costs of €2175 per patient, during the year before receiving LBP Care at the Groningen Spine Center. Furthermore, out of the employed patients 36.4% reported sick days for LBP in the previous 6 months, which resulted in mean costs in terms of productivity loss of €1380 per patient.

Moreover, exercise therapy and education were found to be slightly more effective than no treatment and other conservative treatments in reducing the burden associated with CLBP [9,11,12,13].

## 2. Materials and Methods

### 2.1. Secondary Data Availability in Italy

In the Italian national healthcare field, the collection, processing, and treatment of data relevant to the statutory healthcare system and to citizens’ health status fall within the mandate of the Ministry of Health (MoH). The MoH, in 1984, established the healthcare information system (HIS) specifically to this end. The regions collect data from the Local Health Units (LHAs) of their territory and transmit them to the Health Ministry. Common and interoperable languages have been specifically developed for the National Health Service’s (NHS) sub-components (hospitalization; outpatient specialized treatment; monitoring of care networks; information system on mental health; national information system on addictions; traceability of pharmaceuticals; emergency; home care; residential and semi-residential care; fee exemptions for chronic pathology; and others), allowing these different informatics systems to interact. Consequently, specific data flows with the required information were collected (individual characteristics, treatments/drugs, tariffs, and co-pay fee exemption) generated by citizens’ contacts with the SSN. Patients are identified by the same code across all data flows: only the data provider (LHAs) can link the code to the real identity of patients, according to the general data protection regulations (GDPR) [14].

The availability of existing and standardized secondary data sources shows a clear advantage in terms of administrative burden and costs related to collecting and processing relevant data for informing policies.

In particular, the data above allow one to identify specific disease patients and their comorbidities or to perform predictive analytics (risk stratification, simulation, and mapping), based on their association with specific healthcare process events, by combining different variables from many sources of administrative data and using appropriate rule-based systems [15]. On the basis of different informative purposes, there are several approaches to health risk stratification, and each has advantages and limits. Methods to segment and stratify populations generally use quantitative and/or qualitative data from secondary and primary sources: currently, hybrid approaches seem to be more efficient than using single sources of data [16], but it also depends on the risk stratification purposes (prevention, diagnosis, treatment, or social supply) and the setting with which each rule-based system is more consistent (e.g., specialist settings vs. general practices) [17].

Italian secondary sources of health data also show some limits, e.g., drug consumption is estimated on the basis of prescribed drugs without considering the real compliance rates for the prescribed medication. Moreover, their structure and the timeliness with which they become available mostly reflect their main value for administrative purposes rather than their efficacy in quickly monitoring health and social aspects. 

Regarding social welfare services, secondary data about the elderly, the disabled, and needy families are firstly collected by local authorities and the LHAs. Data are generally handled in aggregate form by different Italian Ministries and are made available for wide consultation by the National Institute of Statistics (ISTAT), grouped by themes and topics. The aggregated structure of these data does not allow them to be linked with other sources of data through primary keys. So, large population-based risk stratification activities, carried out through secondary data sources, are mainly performed referring to the healthcare data only. This makes the identification of primary data sources strategic for including social determinants and further health data dimensions in risk stratification models.

### 2.2. The Participatory Paradigm in Primary Data Collection

Primary data are collected directly from their original source specifically to meet the needs of data collection, and they are usually up-to-date. However, compared to secondary data, primary data collection is generally considered as very expensive and time-consuming, even though many web-based primary data collection tools are currently available to acquire data, while simultaneously increasing the response rate from respondents.

The recent pandemic further promoted the development of mobile-phone-based apps and digital platforms for surveillance and detection of data to aid COVID-19 control [18]. In our opinion, these types of experiences deserve to be further explored within the Italian NRRP strategies.

If modern medicine identifies the key for successful healthcare as the “patient-centered” approach based on predictive, preventive, personalized, and participatory aspects [19], this implies that not only the physician but also the patients evolve their role, shifting from a reactive position to a proactive approach to health, and that this process should be supported by the widespread sharing of information between patients and healthcare providers. To date, when dealing with personal health data, the citizen who is the source of data has very little control over and has little benefit from these data [20].

Participatory disease surveillance is an emerging approach to supplement “traditional” disease surveillance approaches. It consists of collecting data for public health, directly involving the population at risk by means of a variety of survey tools. Moreover, self-management education, which complements traditional patient education in supporting patients to attain the best possible quality of life with their chronic condition, is part of the new chronic disease paradigm: a patient–professional partnership that should be further promoted [21]. In particular, self-management education teaches problem-solving skills [21], so interactive self-management tools for people with chronic conditions would significantly improve their quality of life and their community capacity.

In the social sphere, the rise in healthcare costs for long-term conditions, added to increasing shortages in the healthcare workforce, shifted the burden of primary healthcare to family caregivers [22]. This huge burden has not been balanced by a concrete ability to capture patients’ and their caregivers’ perspectives and an ability to consider that their priorities and needs could be at variance with those of their healthcare providers [23].

### 2.3. Innovative Tools for People Engagement 

Mismatching between complex individual or community needs and health/social supply have to be overcome through people’s engagement in collecting both outcome data for identifying public policies and output data for monitoring their effectiveness. The use of innovative tools for freely collecting big data, exploiting the combined technology offering and its widespread access across all segments of the population, should be the winning strategy. In particular, we refer to smart and free tools specifically designed and developed to establish a lasting link between the user and the tool, for promoting innovative cooperative strategies in social and health fields. These tools should be aimed to: (1) collect primary data by involving directly the population; (2) improve community capacity development, by strengthening the digital skills of people; (3) promote community participation, by involving people in making decisions about their own communities; and (4) direct the citizens towards protective strategies for their own general health. 

Toward this aim, during the SARS-CoV-2 pandemic, we developed the “Doing Risk sElf-assessment and Social health Support” for COVID (Dress-COV) system, based on the Telegram bot [18]. Dress-COV was originally designed and developed to understand, catalogue, and scientifically assess parameters potentially related to the disease process, extending from risk assessment for SARS-CoV-2 infection to the actual disease and outcomes of COVID-19. The Dress IT architecture was also originally developed to allow customized potential reuse in different health and social scenarios by simply identifying the most appropriate survey questions for monitoring specific outputs and outcomes. The most concrete results of using Dress-COV are (1) high compliance with the tool; (2) its potential for territorial health providers; (3) the high timeliness with which user data becomes available; (4) the versatility of the IT architecture that allows continuous updating of survey questions; and (5) the easy export of structured data, to be analyzed by using many statistical tools. Moreover, on May 2021, Italian Telegram users amounted to 13 million people, and they are constantly increasing because of the app’s very high levels of security (end-to-end encryption for chats; possibility to set the self-destruction of messages, videos, and photos) and the app’s other features that are particularly popular among users (no ads, possibility to define wide chat groups and easily share multimedia content). 

Most informative systems or digital products fail in achieving their original purposes for some reasons such as: misplaced priorities, ignorance about real users, conflict of interest, and lack of a design process [24]. This is particularly relevant considering that individual health is a global common good; it cannot be thought of as a private good (it is not consumed, it is not a rival) and requires a global approach and many different sources of data for defining its policies [25]. Facing chronicity and long-term care management is one of the current challenges in health care and producing incremental changes in this field also requires the development of cooperative information systems following a user-centered approach based on a goal-oriented design (GOD) methodology [24], in which end users (citizens at higher risk of chronicity, and patients and their caregivers) and stakeholders (public and private territorial health providers, municipalities, and community organizations) guide the process, as they will ultimately validate the final product.

## 3. Results

Our proposal concerns the development of a cooperative information system for supporting the citizens in the management of CLBP and promoting efficiency of CLBP care plans.

We propose a customization of the Dress system, the Dress-KINESIS, as the base for CLBP cooperative information system implementation. Dress-KINESIS will be able to collect primary data and link it to the existing standardized secondary data sources. The design of Dress-KINESIS will be performed following different phases, as summarized below: 

(1) *Define and validate the IT architecture features*: as the focus of health systems moves toward supporting wellness anytime and anywhere, an open platform environment requires a reference framework around technology to connect and share data.

An optimal platform has to be modular, separating content and technology and being able to incorporate third party systems. This implies a networked infrastructure that could integrate different information from multiple sources. Design considerations should provide clear data provenance to deliver trusted algorithms. The IT architecture, structured in different layers and built on common standards, forms the base for third-party applications, ensuring safe and interoperable systems [26].

Starting from these hypotheses, the cooperative information system will be implemented within the Dress system IT architecture, based on the bot technology offered by the Telegram messaging application. It allows the creation of virtual users that are able to perform actions in an automatic way, collect data, provide answers to user queries, inform people making decisions, and assess risk behaviors in different scenarios by querying users.

**Figure 2:**
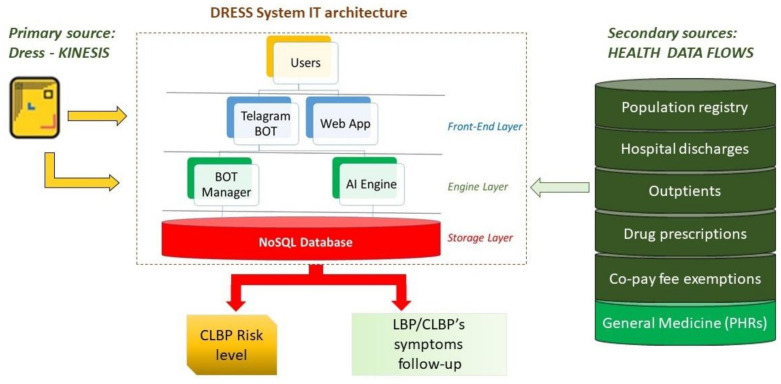
Cooperative information system design.

As shown in Figure 2, Dress IT architecture is composed of three layers: the front-end, the engine, and the storage layer [18].

Relevant information from secondary data sources can be incorporated in the engine layer. In the same location, the management of bot automation and the analysis of the primary data take place, collected through the Dress-KINESIS customization. The engine layer will provide predictive risk models.

In order to validate the Dress-KINESIS, before launching the tool into the live environment, it will be tested on a sample of at least 100 users, according to the goal-oriented design methodology.

(2) *Draw the conceptual reference model*: an informative system about chronicity needs to be developed following a logical scheme, in a cross-temporal manner, from the risk assessment to the syndrome/disease outcome, throughout the dynamic process of taking charge of citizens with higher risk of chronicity or who are already ill. This firstly implies the identification of the most relevant scientific evidence to define the syndrome/disease determinants, features, and impacts; the end users’ needs and focus; the stakeholders who could be engaged; and the different strategies of prevention and care. This is particularly relevant when referring to chronic health needs requiring an efficient distribution of resources across different care sectors (primary care, hospital care, and long-term care) and a particular focus on prevention.

The Ideal Transitions in Care (ITC) framework [27] has been proposed for suggesting best practice to guide new interventions in transitions of care from the hospital to the community and create process measures for monitoring the quality of care transitions. The ITC proposes 10 domains to consider to ensure safe transitions: (1) complete communication of information; (2) availability, timeliness, clarity, and organization of information; (3) medication safety; (4) educating patients to promote self-management; (5) monitoring and managing symptoms after discharge; (6) enlisting help of social and community supports; (7) advanced care planning; (8) coordinating care among team members; (9) discharge planning; and (10) follow-up with outpatient providers. 

Moreover, Burke [27] suggested that appropriate methods to risk-stratify patients at the time of discharge and then selectively apply interventions based on this analysis may maximize efficacy and minimize cost. This approach could also be applied regarding care sectors other than hospitals.

Finally, adherence to care plans for the efficient management of chronic conditions has to be fostered by promoting interprofessional health care, an approach characterized by a high degree of collaboration and communication among health professionals. More recent suggestions concern the inclusion of patients/caregivers in the collaborative team [28].

In the management of chronicity, CLBP is particularly challenging because it is a symptom not a disease and because it may be triggered by a great variety of factors. Changes in lifestyle and in ways of working, such as the intensive use of computers and other technologies at work and at home, have increased sedentariness, a risk factor for chronic and acute low back pain due to muscle weakness. Several other factors related to lifestyle, e.g., obesity, smoking, and hypertension, are known risk factors for CLBP [6]. Psychological and psychosocial factors are other LBP determinants [29].

The prevalence of LBP reported by the general practitioners every year amounts to 7% of their patients [30]. Webb et al [31] stated that, in the UK, 21.3% of men and 24.5% of women reported having back pain for a minimum of one week in a month—9.4% of men had pain classed as being intense (12.7% among women), 7.3% as disabling (10.7% among women), 10.5% as chronic (12.3% among women), and 3.9% classed as all three (6.2% among women). 

Notwithstanding the fact that studies of the last two decades have shown that traditional treatment of LBP, focusing on injections and (bed)rest, may contribute to the chronicization of worse quality of life and devastating individual and macro-economic sequelae, general practices’ management still reveals gross deviations from current LBP guidelines, resulting in likely over-diagnosis with imaging techniques and over-treatment with NSAIDs [32,33].

Diagnostic and therapeutic pathways generally involve many different stakeholders engaged in different strategies of CLPB patient management on the basis of the patient risk level. Surgical modalities and/or appropriate analgesics prescription are handled by public and private health providers. Traditional non-surgical modalities, including a range of manual therapy techniques and exercise treatments, are also handled within gyms and private physiotherapy centers, often without an interdisciplinary and comprehensive approach. More recently, the efficacy of interactive self-management tools has also been tested [34] for promoting “transition coaching” to assist patients across health settings and encourage them to be active in their own care while providing them the necessary tools to do so.

Efficiency in managing CLBP also depends on the possibility to promote collaborative team, including either health professionals, kinesiologists, or patients, in order to define care plans aimed to reduce non-surgical hospitalizations rate, overuse of diagnostic imaging, overprescribing of medications and painkillers, and to improve other non-surgical approaches, which are partly self-manageable, identified according to the risk stratification level of each patient.

**Figure 3:**
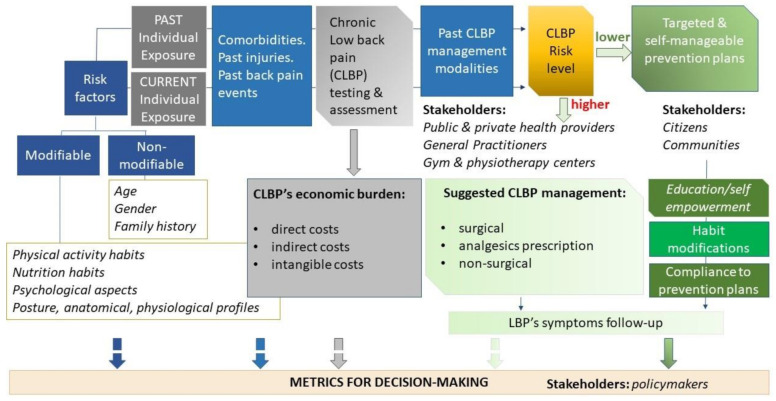
The reference conceptual model proposed for the Dress-KINESIS.

The reference conceptual model proposed for the Dress-KINESIS is shown in Figure 3.

The cooperative information system about CLBP aims to analyze individual data about the Dress-KINESIS users, both that extracted from secondary sources and that provided by the users themselves. Analyses will be performed using the most appropriate machine learning methods, to estimate the risk of symptoms worsening or new onset of symptoms. Furthermore, the risk stratification allows one to return useful information about non-surgical modalities for managing CLBP and self-management programs for improving emotional management (e.g., mindfulness-based stress reduction technique or progressive muscle relaxation) and physical functioning (functional exercises, postures and self-postures, stretching, pilates, and many others). CLPB’s economic burden will also be evaluated, through integration among secondary and primary data. In particular, the main interest concerns indirect and intangible costs estimation, which are the most difficult to provide using secondary data only.

(3) *Identify the existing secondary sources of data*: secondary data sources are mainly available in the healthcare field, but these data have an information potential limited to the fact that they were originally collected for a different purpose. In particular, we refer to the health data flows routinely collected within the Italian Healthcare Information System, but also to the electronic Personal Health Records (PHRs) managed by general practitioners. For each source of data, appropriateness, completeness, information potentialities, limits, and the capability to be linked to other data have to be considered before their use. Integration among different secondary data sources, in particular, allows one to identify patients’ health co-morbidities based on their past contacts with the National Health System and to estimate patients’ healthcare costs, only referring to the tariffs of the hospitalizations, outpatient visits, diagnostics, and medications. Out-of-pocket costs are not routinely collected within the Italian Healthcare Information System. 

To perform a pilot analysis about the information potential of the health data flows routinely collected within the Italian Healthcare Information System, we used an ad hoc database deriving from the linking among hospitalization, outpatient visit, drug prescriptions, and co-payment exemption data, collected by a hospital in Tuscany during the 2011 year. We identified 529 CLBP patients (52% women) and their characteristics (age, gender, and co-morbidity rate). CLBP patients’ healthcare costs have also been estimated, referring to the tariff associated with each treatment: the disease-related group (DRG’s) reimbursements for hospitalization, the tariff rates for outpatient treatments, and the reimbursement price for drug prescriptions. Given a total estimated healthcare cost of over 95,000 euros for women and almost 88,000 euros for men, patients between 35 and 54 years of age showed the highest costs. In particular, among women, the per capita cost is higher than men’s for all types of healthcare costs (hospitalization: 3672 vs. 3023 euros; medications: 139 vs. 47 euros; and outpatient visits and diagnostic: 164 vs. 185 euros). These costs represent only a part of the real overall costs because: (a) hospitalized patients are a very limited percentage of those suffering from CLBP and (b) information about out-of-pocket medications, e.g., the painkiller drugs, and physiotherapy costs, which determine a great economic impact, were not available [10].

(4) *Plan the primary data collection following a participatory method*: limitations in information potential of secondary data can be overcome by implementing primary data collections whose strategy, operationalization of the theoretical construct, and research design can be tailored to the specific research question. Moreover, primary data collections have the potential of putting particular emphasis on health promotion rather than health surveillance only. Many participatory disease surveillance tools are structured around the reporting of syndromic information, that is, self-reported symptoms of illness that are not generally collected within secondary sources of data [35].

Primary data collection is known to be costly and time-consuming, but the current availability of free and smart tools, which are easily shareable, makes primary collection more efficient than in the past. 

The Dress-KINESIS customization, based on the Dress IT architecture, will be developed by identifying the most appropriate survey questions for monitoring specific outputs and outcomes throughout the process of managing CLBP patients. Survey questions will investigate several aspects such as demographic features and familiar history, personal and social habits, past symptoms and/or injuries, and psychological distress. Pain intensity, disability, and physical impairment will also be investigated. Valid scales will be addressed if available, e.g., the Oswestry low back pain disability questionnaire, the Roland–Morris disability scale, the Beck Depression Inventory, the Zung Self-Rated Depression Scale, and others. 

By evaluating the individual experience of CLBP prevention and treatment, the survey questions will also aim to draw the “as is” scenario and to identify several plausible alternative future developments (“to be” scenarios) for increasing efficiency throughout the process of CLBP management and/or promoting innovative approaches such as plausible and personalized self-manageable preventive plans [36].

(5) *Integrate secondary and primary data sources*: within the Italian Healthcare Information System, patients are identified by the same pseudonymized code across all data flows, both in order to allow the linking between different sources of secondary data and ensure personal data protection. Moreover, the PHRs collected by general practitioners can be linked to other secondary sources by using the personal code reported on the Health Insurance Card, a personal document issued to individuals entitled to benefit of the Italian National Health Service [37]. 

Within the primary data sources, each individual is randomly associated with a unique identification code generated following various strategies, highly impacted by the GDPR. This generally makes the joining of primary and secondary sources of data impracticable. 

Even though no system can guarantee absolute data security, the Dress system ensures a balance between gathering data from people while protecting their privacy. Firstly, according to the Art. 7 of the GDPR, citizens who will use the Dress-KINESIS will be asked to sign the consent to process their pseudonymized data. The tool access will be restricted to persons that have provided consent. Secondly, the Telegram user’s Identification Number or ID is a unique number that is randomly associated with each user, and it does not depend on the user’s phone number or associated personal data. Thirdly, linking the data provided by the users to other sources of personal health data will be restricted to users who provided a further explicit consent to being linked to their GPs’ PHRs. Finally, according to the Art. 32(1) of the GDPR, both the GPs’ and the citizens’ private data will be encrypted before their use for scientific purposes.

This strategy protects user’s privacy because it ensures (a) the management of individual sensitive personal data only by those organizations belonging to the NHS (the GPs and the LHUs) and (b) the use of data for scientific purposes in pseudonymized form only. 

Furthermore, from a structural point of view the Dress system is already designed to incorporate third-party sources of data within its engine layer.

(6) *Define and validate the risk assessment process*: the individual risk of developing a specific syndrome/disease, its co-morbidities, and other disease risk factors needs to be considered. Modelling activities can be performed using more traditional (rule-based systems, algorithms) and innovative methodologies (machine learning) to segment and stratify populations [38].

Information gathered in the secondary sources of data and that collected through the Dress-KINESIS will be used to identify the risk level of each individual involved in the CLBP cooperative information system. A series of Python algorithms designed to train AI based models, both supervised and unsupervised, have already been implemented in the Dress system to analyze the acquired data and have been enabled to propose new predictive risk models. The artificial intelligence models will be fully trained and with increasing validation based on the number of users who will join the system. Further validation of the risk profiling system will be provided by sharing with the GPs the information collected through the Dress-KINESIS (baseline data, risk level, and follow-up data) about the GPs’ patients who provided explicit consent. This validation limits the criticisms correlated to a mere algorithmic decision-making process as bias, opacity, and risk of discrimination [39], according to the Articles 4 and 22 and the Recital 71 of the GDPR about profiling.

(7) *Make a self-manageable plan and health messages for the users*: people with greater health-related self-control respond with more empowering strategies to health news. Health-related self-control is driven by health consciousness and health knowledge [40]. Inadequate health literacy or otherwise the lack of ability to sort through online health-related information could lead to misinterpretation or misuse of information by patients [41]. Strategies for eHealth literacy assessments accompanied by targeted resources that point individuals to high-quality and credible health information should be performed.

Dress-KINESIS survey questions and suggested self-manageable plans will be identified by reviewing the most relevant scientific references about LBP assessment and management, referring to the biopsychosocial model for investigating behavioral, psychological, and social factors associated with CLBP.

Targeted feedback will be implemented based on the individual risk of the users and will be adapted to be easily understandable. Self-manageable plans will also be shared using images, because they have a significant impact on people’s non-conscious (unintentional) and reflective (intentional) responses to the proposed exercises [42,43,44,45,46].

A multidisciplinary core team of epidemiologists, kinesiologists, physiotherapists, psychologists, osteopaths and clinicians with proven expertise in the LBP domain and survey methods will be involved. The core team will be supported by bioengineering expertise in developing intuitive and easy-to-use functions to be implemented within the tool and IT expertise in designing the cooperative information system.

(8) *Plan the metrics for decision-making*: based on the understanding that wasteful spending is still widely prevalent in several segments of modern healthcare systems, improving efficiency is an objective of first-order importance for health policy-makers. Efficiency in the healthcare field is described as the ratio between health system inputs and either outputs or health outcomes. In particular, allocative efficiency addresses the issue of deploying the right mix of outputs that maximizes welfare according to societal preferences. Identifying the right mix of outputs requires knowledge of the relative value of different health system outputs attributed by citizens. 

At the provider level, considering a patient’s experience where the inputs are the resources spent for the treatment (and prevention) of a disease and the output is the resulting health gain, the allocatively efficient decision is to treat the patient with the most cost-effective treatment available. Moreover, a provider would thus be ‘allocatively efficient’ if its prevention or treatment recommendations complied with clinical guidelines, assuming those had been developed to reflect cost-effectiveness.

At the system level, allocative efficiency is reached when resources are distributed across different care sectors (e.g., prevention, primary care, hospital care, and long-term care) in such a way that the mixture of care services provided maximizes the aggregate health gain produced by the healthcare system as a whole.

Considering that health outcomes are also influenced by several non-health care determinants (exposure to lifestyle-related risk factors and non-lifestyle related risk factors), allocative efficiency can also be conceptualized at the societal level, considering other areas of health-producing welfare, such as social protection or education, whose spending impacts on the amount of resources devoted to the production of health care. 

Lowering the risk of developing a disease through specific strategies for promoting community participation, allowing widespread sharing of information between patients and healthcare providers, and fostering self-management education can be considered as outcome indicators at the system, provider, and citizen levels.

## 4. Discussion

Starting from an awareness of the actual mismatch between complex community needs and health/social supply, this paper aims to support the current discussion about the Italian National Recovery and Resilience Plan in its purpose of innovation in governance and services by highlighting the strategic role of building information systems based on the link between secondary sources of health data and primary data, collected following a participatory strategy and using innovative tools. 

From this perspective, this work proposes a multi-phase process for the design of a cooperative information system for supporting citizens in the management of CLBP and estimating a set of indicators about the chronic conditions considered as the first cause of disability and driver of a significant economic burden. 

In achieving these goals, we referred to the best practices suggested within the Ideal Transitions in Care framework [27] for fostering the transition of care from the hospital to the community, and we exploited social capital (high degree of collaboration and communication among different public and private stakeholders) for promoting cooperative development, highlighting the potential of health data cooperatives [20] and identifying appropriate metrics for monitoring quality-of-care transitions. 

We also referred to the European Commission inference on Health System Performance Assessment [47], which indicates several opportunities for European countries to improve their tools and methodologies for measuring and assessing efficiency of care. In particular, the suggested strategies include: (1) increasing the quality and granularity of cost data; (2) using a disease-based approach to benchmark efficiency of care provided in the whole care cycle; (3) expanding efficiency measurement to other care sectors as primary, mental, and long-term care for retaining greater control of the ‘non-hospital’ factors that cause unnecessary use of hospitals; and (4) customizing communication about efficiency of health care based on the ‘capacity to react’ of different audiences (health managers, policymakers, citizens, and clinicians). 

The Italian NRRP is developed around three strategic axes shared at a European level: digitisation and innovation, ecological transition, and social inclusion. In particular, within the sixth mission (healthcare), it proposes to strengthen local prevention and health services, modernizing and digitizing the health system and ensuring equal access to care [3]. This can be achieved by leveraging knowledge sharing among public/private scientific entities to improve applied research for translating scientific findings into prevention or potential treatments for disease and for redesigning pathways to provide health services.

Italian NRRP also promotes the skill mix change between clinicians and other healthcare professionals by identifying new roles and skills in taking charge of patients and their complexity, as well as providing for the development of new technological self-monitoring tools and cooperative strategies. Moreover, the NRRP aims to provide more effective information systems for ensuring the completion and dissemination of the Electronic Health Records (EHRs) and the better delivery and monitoring capacity of the Essential Levels of Assistance (LEA).

Furthermore, the NRRP aims to promote the transition of chronic-care management from the hospital to the community, by strengthening home care services, through the more widespread use of the digital health tools, and by supporting general practices [3].

In our opinion, the proposed cooperative information system about CLBP, based on an innovative approach to collecting primary data that are able to be linked to secondary data, meets the European strategies proposed above and the main PRRN aims.

It is designed considering the metrics for measuring efficiency both at the micro and macro levels by increasing the completeness and granularity of cost data, monitoring how the resources are distributed across different care sectors, and suggesting how to improve care plan efficiency based on a patient’s risk stratification. Moreover, the system itself shows its efficacy based on the advantageous rate between its input (the integration among available secondary data sources and primary data) and output (the probability of estimating outcome and efficiency indicators for different pathology profiles, care sectors, and processes).

These features are especially relevant when referring to those health needs requiring a shift of focus from disease treatment to disability prevention/chronic disease management, as happens with CLBP.

In particular, this promotes the capacity of the different stakeholders involved in CLBP management to actively react, by redesigning the pathways to provide health services through the availability of a set of indicators referring to the entire process (a) from risk-factor identification to risk-level estimation, (b) from disease onset to resulting health gain or worsening, and (c) from the choice of the management strategies to the resulting outputs (efficiency) or health outcomes (effectiveness). The participatory approach characterizing the system may also promote the widespread sharing of information between patients and healthcare providers within the framework of a new patient–professional partnership and improve citizens’ self-management education.

Moreover, the project team composition will ensure a skill mix change among individuals with different expertise by integrating researchers from many areas (epidemiologists, kinesiologists, statisticians, bioengineers, and IT developers) with a team of healthcare professionals (physiotherapists, psychologists, osteopaths and clinicians) with proven expertise in the LBP domain. Skill mix change will be also promoted by sharing the information collected through the Dress-KINESIS with GPs, during the validation phase of the risk profiling algorithms.

The project team composition, the nature of data collected (real patient data), and the timeliness with which they become available will also ensure the capability to translate scientific findings into concrete and well-timed indications for healthcare planning and monitoring.

Furthermore, the proposed system, being composed of three main components—(1) the “core” for data acquisition, integration, and secure data management; (2) the front-end layer to provide meaningful user experience; and (3) the “Big Data” analytics system, which can be all customized—has the potential to be adapted to other health questions and scenarios.

Finally, our model could be easily proposed in those EU countries that have universal access to health care and where secondary standardized health data is available and routinely collected (e.g., Italy, France, Denmark, Finland, and Spain). In those countries where health services provision is fully or partly managed by insurance (public, no-profit, or private), the customization of our model is limited by the availability of secondary data and is potentially appliable to subgroups of population only (insurance users).

Currently, the most relevant limitations that we envisage in the proposed system concern the effects of eventual GDPR updates that could be introduced. In particular, we refer to the impact on the: (a) capability of linking secondary and primary data and (b) use of AI techniques for profiling individual citizen’s risk.

The dissemination of the Dress-KINESIS tool could also be a potential critical issue. Efficient communication will have to be planned, and a communication expert will need to be included in the project team. Specific resources in the project budget will need to be collected/allocated. 

## 5. Conclusions 

The proposed system is a feasibility study based on innovative strategies and tools. We are aware that its implementation will entail some difficulties that we cannot foresee now; however, we believe that the value of the system design supported by the multidisciplinary project team expertise will meet the implementation needs in the next two years. The Dress-KINESIS tool has been proposed for different funding calls whose evalutations are still in progress.

## Data Availability

No new data were created or analyzed in this study. Data sharing is not applicable to this article.

## References

[1] Hartley J. (2005). Innovation in Governance and Public Services: Past and Present. Public Money Manag..

[2] Schumann A. (2016). Using Outcome Indicators to Improve Policies: Methods, Design Strategies and Implementation.

[3] Ministero delle Finanze The National Recovery and Resilience Plan (NRRP). https://italiadomani.gov.it/en/home.htmlh.

[4] Benington J., Hartley J. Pilots, paradigms and paradoxes: Changes in Public Sector Governance and Management in the UK. Proceedings of the International Research Symposium on Public Sector Management.

[5] Greenhalgh T., Robert G., Bate P., Kyriakadou O., MacFarlane F., Peacock R. (2004). How to Spread Good Ideas.

[6] Bento T.P.F., Genebra C.V.D.S., Maciel N.M., Cornelio G.P., Simeão S.F.A.P., de Vitta A. (2019). Low back pain and some associated factors: Is there any difference between genders?. Braz. J. Phys. Ther..

[7] Hoy D., March L., Brooks P., Blyth F., Woolf A., Bain C., Williams G., Smith E., Vos T., Barendregt J. (2014). The global burden of low back pain: Estimates from the Global Burden of Disease 2010 study. Ann. Rheum. Dis..

[8] Walker B.F., Muller R., Grant W.D. (2004). Low Back Pain in Australian Adults. Prevalence and Associated Disability. J. Manip. Physiol. Ther..

[9] de Campos T.F., Maher C.G., Fuller J.T., Steffens D., Attwell S., Hancock M. (2020). Prevention strategies to reduce future impact of low back pain: A systematic review and meta-analysis. Br. J. Sports Med..

[10] Dutmer A.L., Preuper H.R.S., Soer R., Brouwer S., Bültmann U., Dijkstra P.U., Coppes M.H., Stegeman P., Buskens E., van Asselt A.D. (2019). Personal and Societal Impact of Low Back Pain. Spine.

[11] Hayden J., van Tulder M., Malmivaara A., Koes B. (2005). Exercise therapy for treatment of non-specific low back pain. Cochrane Database Syst. Rev..

[12] Choi B.K., Verbeek J.H., Tam W.W.-S., Jiang J.Y. (2010). Exercises for prevention of recurrences of low-back pain. Cochrane Database Syst. Rev..

[13] Chaleat-Valayer E., Denis A., Abelin-Genevois K., Zelmar A., Siani-Trebern F., Touzet S., Bergeret A., Colin C., Fassier J.-B. (2016). Long-term effectiveness of an educational and physical intervention for preventing low-back pain recurrence: A randomized controlled trial. Scand. J. Work. Environ. Health.

[14] GDPR Regolamento 2016/679. https://www.garanteprivacy.it/regolamentoue.

[15] Hripcsak G., Albers D.J. (2013). Correlating electronic health record concepts with healthcare process events. J. Am. Med. Inform. Assoc..

[16] Jean-Baptiste D., O’Malley A.S., Shah T. (2017). Population Segmentation and Tailoring of Health Care Resources: Findings from a Literature Review.

[17] Franchini M., Pieroni S., Passino C., Emdin M., Molinaro S. (2018). The CARPEDIEM Algorithm: A Rule-Based System for Identifying Heart Failure Phenotype with a Precision Public Health Approach. Front. Public Health.

[18] Franchini M., Pieroni S., Martini N., Ripoli A., Chiappino D., Denoth F., Liebman M.N., Molinaro S., Della Latta D. (2020). Shifting the Paradigm: The Dress-COV Telegram Bot as a Tool for Participatory Medicine. Int. J. Environ. Res. Public Health.

[19] Lejbkowicz I., Caspi O., Miller A. (2012). Participatory medicine and patient empowerment towards personalized healthcare in multiple sclerosis. Expert Rev. Neurother..

[20] Kossmann D., Brand A., Hafen E. (2014). Health Data Cooperatives—Citizen Empowerment. Methods Inf. Med..

[21] Bodenheimer T., Lorig K., Holman H., Grumbach K. (2002). Patient Self-management of Chronic Disease in Primary Care. JAMA.

[22] Gibbons M.C., Shaikh Y., Weaver C., Ball M., Kim G., Kiel J. (2016). The Patient of the Future: Participatory Medicine and Enabling Technologies. Healthcare Information Management Systems.

[23] Upshur R.E., Tracy S. (2008). Chronicity and complexity: Is what’s good for the diseases always good for the patients?. Can. Fam. Phys..

[24] Cooper A., Reimann R., Cronin D., Noessel C. (2014). About Face: The Essentials of Interaction Design.

[25] Soucat A., Kickbusch I. (2020). Global Common Goods for Health: Towards a New Framework for Global Financing. Glob. Policy.

[26] Roberts D., McBride A., Dunscombe R. How Will You Design Information Architecture to Unlock the Power of Data? Creating the Right Data Environment for a Connected Health Ecosystem. EY 2020 Report on Global Health Technology. https://assets.ey.com/content/dam/ey-sites/ey-com/en_gl/topics/health/ey-global-health-tech.pdf.

[27] Burke R.E., Guo R., Prochazka A.V., Misky G.J. (2014). Identifying keys to success in reducing readmissions using the ideal transitions in care framework. BMC Health Serv. Res..

[28] Pascucci D., Riccardi M.T., Sapienza M., Nurchis M.C., Ricciardi W., Morano C., Damiani G. (2020). Interprofessional collaboration and chronicity management: A systematic review of clinical trials. Eur. J. Public Health.

[29] Azfar S.M., Murad M., Azim S.R., Baig M. (2019). Frequency of and Various Factors Associated with Stress, Anxiety, and Depression among Low Back Pain Patients. Cureus.

[30] Parsons S., Ingram M., Clarke-Cornwell A., Symmons D. (2011). A Heavy Burden: The Occurrence and Impact of Musculoskeletal Conditions in the United Kingdom Today.

[31] Webb R., Brammah T., Lunt M., Urwin M., Allison T., Symmons D. (2003). Prevalence and Predictors of Intense, Chronic, and Disabling Neck and Back Pain in the UK General Population. Spine.

[32] Piccoliori G., Engl A., Gatterer D., Sessa E., Der Schmitten J.I., Abholz H.-H. (2013). Management of low back pain in general practice—Is it of acceptable quality: An observational study among 25 general practices in South Tyrol (Italy). BMC Fam. Pr..

[33] Meroni R., Piscitelli D., Ravasio C., Vanti C., Bertozzi L., De Vito G., Perin C., Guccione A.A., Cerri C.G., Pillastrini P. (2021). Evidence for managing chronic low back pain in primary care: A review of recommendations from high-quality clinical practice guidelines. Disabil. Rehabil..

[34] Chiauzzi E., Pujol L.A., Wood M.M., Bond M.K., Black R., Yiu B.E., Zacharoff K. (2010). painACTION-Back Pain: A Self-Management Website for People with Chronic Back Pain. Pain Med..

[35] Smolinski M.S., Crawley A.W., Olsen J.M., Jayaraman T., Libel M. (2017). Participatory Disease Surveillance: Engaging Communities Directly in Reporting, Monitoring, and Responding to Health Threats. JMIR Public Health Surveill..

[36] Vollmar H.C., Ostermann T., Redaèlli M. (2015). Using the scenario method in the context of health and health care—A scoping review. BMC Med Res. Methodol..

[37] Official Site of the Agenzia delle Entrate Health Card. https://www.agenziaentrate.gov.it/portale/web/english/nse/individuals/health-card.

[38] Franchini M., Pieroni S., Cutilli A., Caiolfa M., Naldoni S., Molinaro S. (2019). The Individual Profile of Pathology as a New Model for Filling Knowledge Gaps in Health Policies for Chronicity. Front. Med..

[39] Castets-Renard C. Accountability of Algorithms in the GDPR and Beyond: A European Legal Framework on Automated Decision-Making. https://ir.lawnet.fordham.edu/iplj/vol30/iss1/3.

[40] Chang C. (2019). Self-Control-Centered Empowerment Model: Health Consciousness and Health Knowledge as Drivers of Empowerment-Seeking through Health Communication. Health Commun..

[41] Silver M.P. (2015). Patient Perspectives on Online Health Information and Communication With Doctors: A Qualitative Study of Patients 50 Years Old and Over. J. Med. Internet Res..

[42] Cope K., Vandelanotte C., Short C., Conroy D.E., Rhodes R., Jackson B., Dimmock J., Rebar A.L. (2018). Reflective and Non-conscious Responses to Exercise Images. Front. Psychol..

[43] Palazzo C., Klinger E., Dorner V., Kadri A., Thierry O., Boumenir Y., Martin W., Poiraudeau S., Ville I. (2016). Barriers to home-based exercise program adherence with chronic low back pain: Patient expectations regarding new technologies. Ann. Phys. Rehabil. Med..

[44] Antoniewicz F., Brand R. (2016). Learning to Like Exercising: Evaluative Conditioning Changes Automatic Evaluations of Exercising and Influences Subsequent Exercising Behavior. J. Sport Exerc. Psychol..

[45] Markland D., Hall C.R., Duncan L.R., Simatovic J. (2015). The effects of an imagery intervention on implicit and explicit exercise attitudes. Psychol. Sport Exerc..

[46] Medina-Mirapeix F., Escolar-Reina P., Gascón-Cánovas J.J., Montilla-Herrador J., Jimeno-Serrano F.J., Collins S.M. (2009). Predictive factors of adherence to frequency and duration components in home exercise programs for neck and low back pain: An observational study. BMC Musculoskelet. Disord..

[47] European Commission (2019). Tools and Methodologies to Assess the Efficiency of Health Care Services in Europe—An Overview of Current Approaches and Opportunities for Improvement.

